# Differential Effect of P2X7 Receptors on Proliferation and Migration of Human Keratinocytes and Dermal Fibroblasts

**DOI:** 10.3390/ijms26178548

**Published:** 2025-09-03

**Authors:** Marta Soszyńska, Michał Komorowski, Krzysztof Łuszczyński, Marcin Radziszewski, Natalia Krześniak, Katerina Shevchenko, Dariusz C. Górecki, Jacek Malejczyk, Aneta Ścieżyńska

**Affiliations:** 1 Department of Histology and Embryology, Medical University of Warsaw, 02-004 Warsaw, Poland; marta.soszynska@wum.edu.pl (M.S.); michal.komorowski@wum.edu.pl (M.K.); kluszczynski@wim.mil.pl (K.Ł.); marcin.radziszewski@wum.edu.pl (M.R.); katerina.shevchenko@wum.edu.pl (K.S.); jacek.malejczyk@wum.edu.pl (J.M.); 2 Laboratory of Molecular Oncology and Innovative Therapies, Military Institute of Medicine National Research Institute, 128 Szaserów Street, 04-141 Warsaw, Poland; 3 Department of Thoracic Surgery, National Medical Institute of the Ministry of the Interior and Administration, 02-507 Warsaw, Poland; 4 Department of Plastic and Reconstructive Surgery, Medical Centre of Postgraduate Education, Prof. W. Orlowski Memorial Hospital, 231 Czerniakowska Street, 00-416 Warsaw, Poland; 5 School of Medicine, Pharmacy and Biomedical Sciences, University of Portsmouth, St Michael Bld, White Swan Road, Portsmouth PO1 2DT, UK; darek.gorecki@port.ac.uk; 6 Institute of Health Sciences, Faculty of Medical and Health Sciences, University of Siedlce, 08-110 Siedlce, Poland

**Keywords:** skin, purinergic receptor, P2X7, inflammation, keratinocytes, dermal fibroblasts, wound healing

## Abstract

Purinergic P2X7 receptors are involved in cellular processes such as inflammation, proliferation, and tissue remodeling, although their significance in human skin physiology remains poorly understood. In this study, we demonstrated strong P2X7 receptor immunoreactivity in the basal and granular layers of the epidermis. Cutaneous expression of P2X7 receptors was further confirmed at the level of specific mRNA and protein in cultured primary human keratinocytes and dermal fibroblasts. To reveal a possible role of these receptors in regulation of keratinocyte and fibroblast function, the cells were treated with a P2X7 agonist BzATP, or its selective antagonist A438079. Cell proliferation and viability were assessed using an immunofluorescence-based cell counter, and cell migration was evaluated by wound healing assay. P2X7 stimulation with BzATP significantly inhibited keratinocyte proliferation and migration, while P2X7 inhibition with A438079 significantly enhanced keratinocyte migration. In contrast, fibroblasts displayed minimal response to either treatment. These findings indicate that P2X7 regulates keratinocyte growth, and purinergic signaling may play a role in the skin. Our data also suggest that selective P2X7 inhibition may support re-epithelialization under conditions associated with impaired wound healing.

## 1. Introduction

Wound healing is a dynamic process comprising the stages of hemostasis, inflammation, re-epithelialization, and remodeling, during which various molecular pathways—such as cell stimulation, proliferation, and migration—are activated [1]. Keratinocytes and fibroblasts are the major skin cell types that respond to these stimulatory signals following skin injury [2]. These responses may be triggered by endogenous molecules released into the extracellular milieu by damaged host cells or by activated immune cells [3]. Such molecules, known as alarmins, include S100 proteins, heat shock proteins, interleukin-1 family members, nucleotides, and others [4]. Among nucleotides, adenosine 5′-diphosphate (ADP) and adenosine 5′-triphosphate (ATP) are considered the most important extracellular signaling molecules. Indeed, following tissue injury, high levels of ATP are rapidly released into the extracellular environment [5]. Under basal conditions, ATP is present at concentrations of approximately 1 mM intracellularly and 1 nM–1 µM extracellularly. Upon in vitro cell monolayer scratching, approximately 100 µM of ATP is released from damaged cells into the culture medium [6]. Such elevated concentrations occur only in damaged tissues; therefore, extracellular ATP (eATP) functions as a potent damage-associated molecular pattern (DAMP), actively involved in both innate and adaptive immune responses via purinergic P2X7 receptors.

Purines are endogenous ligands for membrane-bound purinergic receptors, which mediate extracellular communication in a process referred to as purinergic signaling. These receptors are divided into P1 adenosine receptors (with four subtypes), P2X ionotropic nucleotide receptors (seven subtypes), and P2Y metabotropic nucleotide receptors (eight subtypes) [7]. P2X receptors are highly conserved trimeric membrane proteins that function as sodium, potassium, and calcium ion channels gated by extracellular ATP [8,9,10].

P2X receptors are widely expressed in various cells and tissues, where they may play diverse functions. They can operate via short-term or long-term purinergic signaling pathways [11]. Short-term signaling is typical of smooth muscle cells, where P2X receptors mediate fast excitatory junction potentials, and in the central nervous system, where they modulate neuromodulatory responses [9]. In contrast, long-term (trophic) purinergic signaling may influence cell proliferation, differentiation, and motility, playing a central role in the physiological turnover of continuously regenerating cells [11].

The P2X7 receptor is unique among the P2X family in requiring very high eATP concentrations for full activation [12]. Moreover, prolonged activation of P2X7 may induce membrane permeabilization and subsequent cell death, classifying it as a cytolytic receptor [12]. However, the specific role of P2X7 differs across tissues and cell types; its activation can induce pro-apoptotic signaling or promote cell growth, depending on the context.

The role of P2X7 in the skin remains incompletely understood. P2X7 expression has been reported in the epidermis [13]. In murine models, expression of the receptor was found to be diminished in keratinocytes located at the wound edge [14]. In humans, P2X7 is highly expressed in the epidermis, particularly in the upper keratinized and exfoliated layers, suggesting its involvement in keratinization processes [15,16]. Nevertheless, the significance of P2X7 in human skin physiology and wound healing is unclear. The current work was aimed to examine P2X7 expression and the effects of its agonist and antagonist compounds in cultured human keratinocytes and dermal fibroblasts—another cell type involved in the wound healing. Understanding the time-dependent effects of P2X7 signaling in skin cells might offer a molecular basis for treatment methods aimed at inflammatory skin conditions characterized by decreased keratinocyte proliferation or dysregulated wound healing.

## 2. Results

### 2.1. P2X7 Is Expressed in All Layers of the Human Epidermis

**Figure 1:**
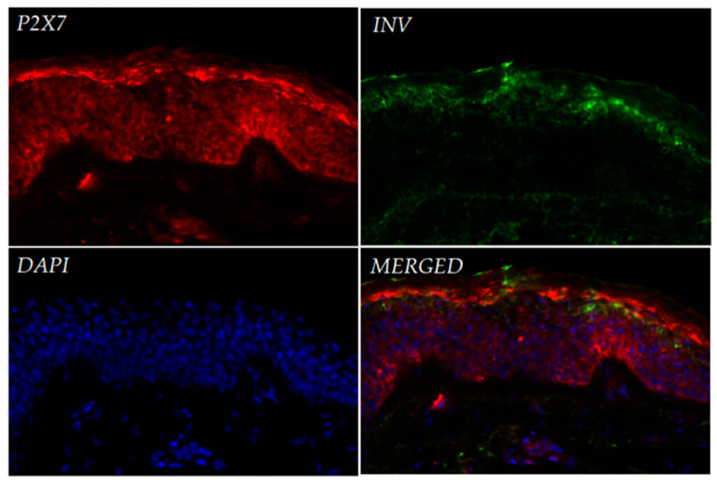
Expression of the P2X7 protein in normal human skin. Cryosections were stained with antibodies against P2X7 (red fluorescence) and involucrin (INV, green fluorescence). Nuclei were counterstained with DAPI (blue fluorescence). Merged images indicate the localization of P2X7 predominantly in the stratum granulosum and basal layers of the epidermis.

Immunofluorescence analysis of P2X7 distribution in normal human skin revealed its expression predominantly within the epidermis (Figure 1). Strong signal intensity was observed in the stratum granulosum, with additional expression detectable in the basal layer. In the dermis, only weak and sparse P2X7 staining was observed. Co-staining with involucrin, a marker of suprabasal keratinocytes, showed partial overlap with the P2X7 signal in upper epidermal layers. DAPI nuclear staining demonstrated intact epidermal structure, with clear layer stratification.

**Figure 2:**
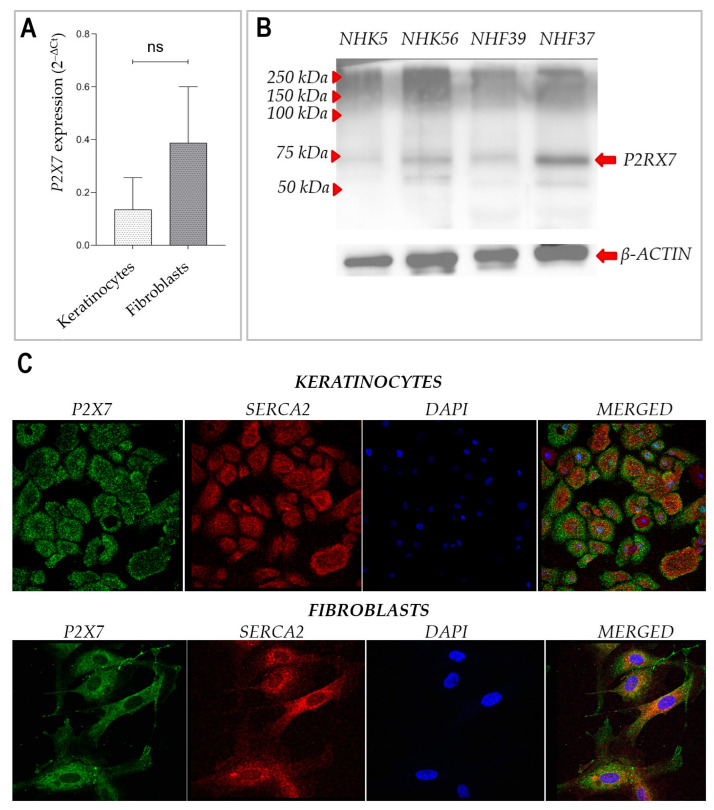
Expression of the P2X7 gene in cultured keratinocytes and fibroblasts isolated from normal human skin. (**A**) Relative P2X7 mRNA expression as assessed by qRT-PCR (*n* = 6); results are expressed as 2^−ΔCt^. Statistical comparison between matched keratinocyte and fibroblast samples was performed using the Wilcoxon matched-pairs signed-rank test. *p* < 0.05 was considered statistically significant. (**B**) Representative Western blot showing P2X7 and β-actin (loading control) in two keratinocyte (NHK5 and NHK56) and two fibroblast (NHF39 and NHF37) primary cell lines. (**C**) Immunofluorescence staining of cultured keratinocytes and fibroblasts with antibodies against P2X7 (green), SERCA2 (red), and DAPI (blue). Merged images show distinct localization patterns in both cell types.

Expression of P2X7 at the mRNA level was confirmed in primary human keratinocytes and dermal fibroblasts, as shown by qRT-PCR (Figure 2A). Although the average relative expression appeared higher in fibroblasts than in keratinocytes, the difference did not reach statistical significance (n.s., *p* > 0.05). Protein expression was assessed by Western blot (Figure 2B), which showed a specific band at approximately 75 kDa, corresponding to the expected molecular weight of P2X7, in all analyzed cell lines. No marked differences in band intensity were observed between keratinocytes and fibroblasts. Immunofluorescence staining of cultured keratinocytes and fibroblasts confirmed the presence of P2X7 protein at the cellular level (Figure 2C). P2X7 staining exhibited both membrane-associated and cytoplasmic localization. Staining did not co-localize with SERCA2, a marker of the endoplasmic reticulum, indicating a distinct subcellular distribution. In keratinocytes, P2X7 staining appeared relatively punctate and peripheral, whereas in fibroblasts, it was more evenly distributed throughout the cytoplasm. DAPI staining demonstrated normal nuclear morphology in both cell types.

### 2.2. Effect of P2X7 Receptor Stimulation on Keratinocyte and Fibroblast Growth Rate

**Figure 3:**
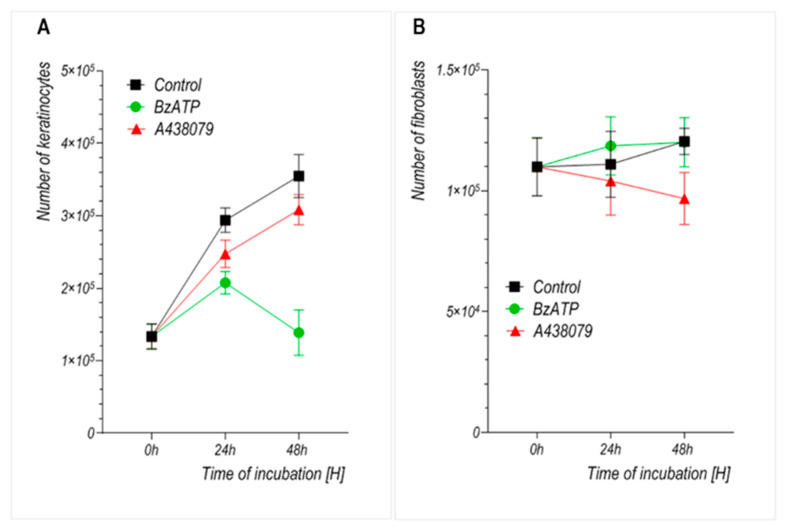
Effect of the P2X7 receptor agonist BzATP and antagonist A438079 on the growth rate of cultured human (**A**) keratinocytes and (**B**) dermal fibroblasts. Results represent mean ± SD from 4 independent experiments. Statistical analysis was performed using one-way ANOVA with Tukey’s post hoc test. Each time point was analyzed separately. *p* < 0.05 was considered significant.

To determine the effect of P2X7 receptor stimulation on the growth of cultured keratinocytes and dermal fibroblasts, the cells were treated with the P2X7 agonist BzATP or the selective antagonist A438079. The number of cells was assessed after 24 and 48 h of incubation. As shown in Figure 3A, BzATP treatment significantly inhibited keratinocyte proliferation, resulting in a 29.4% reduction after 24 h and a 60.9% reduction after 48 h compared to untreated controls (*p* < 0.0001). In contrast, A438079 did not significantly affect keratinocyte proliferation. In dermal fibroblasts, neither BzATP nor A438079 exerted a significant effect on cell proliferation (Figure 3B). It should be noted that the proliferation rate of fibroblasts under basal conditions was substantially lower than that of keratinocytes.

**Figure 4:**
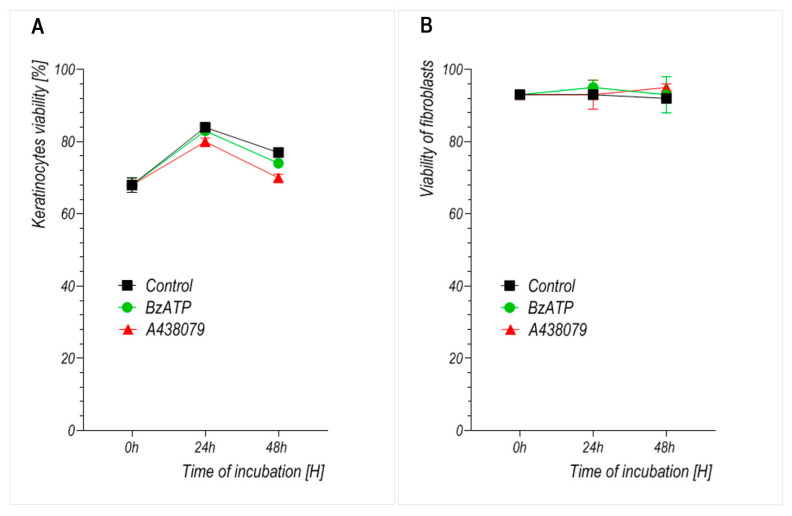
Effect of the P2X7 receptor agonist BzATP and antagonist A438079 on the viability of cultured human (**A**) keratinocytes and (**B**) dermal fibroblasts. Results represent mean ± SD from 4 independent experiments. Statistical analysis was performed using one-way ANOVA with Tukey’s post hoc test. Each time point was analyzed separately. *p* < 0.05 was considered significant.

To exclude the possibility that the observed effects on cell number were due to cytotoxicity, cell viability was assessed (Figure 4). The viability of both keratinocytes and fibroblasts remained high (>70% in keratinocytes and >90% in fibroblasts) across all treatment conditions and time points, with no statistically significant differences between treated and control groups.

### 2.3. Effect of P2X7 Receptor Stimulation on Keratinocyte and Fibroblast Migration

**Figure 5:**
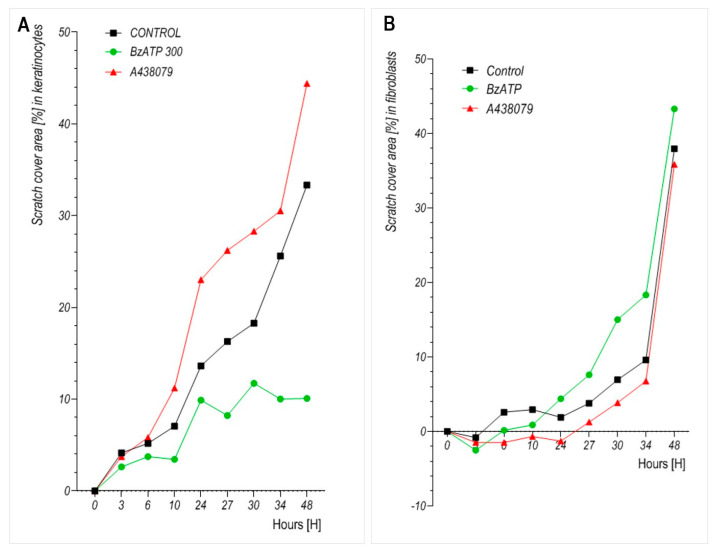
Effect of the P2X7 receptor agonist BzATP and antagonist A438079 on the migration of cultured human (**A**) keratinocytes and (**B**) dermal fibroblasts assessed by wound healing assay. Results represent mean ± SD from 4 independent experiments. Statistical analysis was performed using one-way ANOVA with Tukey’s post hoc test. Each time point was analyzed separately. *p* < 0.05 was considered significant. Error bars were omitted to improve clarity due to high variability in insert-defined gap closure timing across replicates.

The influence of P2X7 receptor modulation on cell motility was evaluated using a cell wound healing assay. Keratinocytes and dermal fibroblasts were cultured with BzATP or A438079, and insert-defined gap closure was monitored over time. As presented in Figure 5A, BzATP markedly reduced keratinocyte migration. The closure area in the BzATP-treated group reached only ~12% after 34 h, whereas the untreated control cells reached ~30%. Conversely, treatment with A438079 enhanced keratinocyte migration compared to the controls, with the closure area exceeding 30% at the 34 h mark. In dermal fibroblasts (Figure 5B), BzATP moderately increased migration compared to control, while A438079 appeared to suppress migration, although the effects were less pronounced than in keratinocytes. Notably, fibroblasts showed delayed closure kinetics of wound area compared to keratinocytes, with migration starting to accelerate after 24 h of incubation.

## 3. Discussion

Immunofluorescence analysis of healthy human skin confirmed P2X7 expression across all layers of the epidermis, with strongest signals observed in the stratum granulosum and basal layers. Our observations are consistent with immunohistochemical data from the Human Protein Atlas, which demonstrate variable expression of the P2X7 receptor across different layers of the epidermis. Notably, in skin samples from older individuals, P2X7 expression appears more prominent in the upper epidermal layers, whereas in samples from younger donors, staining is primarily restricted to the basal and suprabasal layers. The donors in our study were women aged 35–50 years, which may account for the intermediate expression pattern observed in our sections. While this trend requires further validation, it reflects the heterogeneous distribution of P2X7 in human skin and supports earlier findings that suggest its involvement in both keratinized and non-keratinized epithelia, as well as in keratinocyte differentiation and cornification [14,15,17]. Our observations also extend previous findings by demonstrating clear expression of P2X7 mRNA and protein in both cultured primary keratinocytes and fibroblasts. These data confirm that both major cell types in the skin express functional P2X7, with a trend toward higher mRNA expression in fibroblasts, although this difference was not statistically significant.

We observed that prolonged stimulation with BzATP significantly reduced the growth rate of primary human keratinocytes without compromising their viability. This suggests that the inhibitory effect results primarily from reduced proliferation rather than cytotoxicity. Fibroblasts, by contrast, did not exhibit a significant change in growth upon BzATP treatment, which may reflect their intrinsically lower proliferation rate and/or reduced P2X7 sensitivity under these conditions. This interpretation is further supported by the well-established observation that primary dermal fibroblasts exhibit significantly slower proliferation rates than keratinocytes in vitro, with typical doubling times exceeding 48–72 h, while keratinocytes divide more rapidly under standard culture conditions [18,19]. These findings are consistent with previous reports showing that P2X7 may mediate growth inhibition or apoptosis predominantly in rapidly dividing cell types, such as keratinocytes and cancer lines, but not in quiescent or slowly proliferating populations [17,20]. Interestingly, the well-known selective P2X7 antagonist A438079 also attenuated keratinocyte proliferation over time, but less potently than BzATP, while having minimal or even some inhibitory effect on fibroblasts. The finding that both stimulation and inhibition of P2X7 can suppress keratinocyte proliferation suggests that the downstream effects of this receptor may depend not only on its activation state but also on the duration of ligand exposure. This could be due to changes in how the compounds bind to the receptor A438079 and how other inhibitors, such as AZ10606120 or A740003, attach to slightly different regions of the P2X7 structure, which could explain why their actions are not identical [21,22].

Migration assays revealed that BzATP significantly reduced the motility of keratinocytes, which is consistent with previous studies indicating that P2X7 signaling influences epithelial wound closure in both murine and human models [22,23,24]. To assess whether the reduced insert-defined gap closure could be attributed to impaired cell proliferation, we performed parallel cell counting and viability assays under identical experimental conditions (Figure 3 and Figure 4). These analyses confirmed that BzATP markedly suppressed keratinocyte proliferation without affecting cell viability. Taken together, the results suggest that the delayed wound closure observed in BzATP-treated keratinocytes likely results from a combination of diminished proliferation and impaired migration, indicating that P2X7 activation may inhibit insert-defined gap closure. To precisely determine whether BzATP affects migration independently of proliferation, mitomycin C could be used to block cell division. However, in our study, we intentionally avoided additional pharmacological interference to preserve the natural behavior of cultured cells and to evaluate the overall long-term effect of BzATP on insert-defined gap closure. Interestingly, P2X7 inhibition via A438079 clearly enhanced keratinocyte migration, with an accelerated insert-defined gap closure observed after 24 h. Since A438079 treatment did not affect keratinocyte proliferation (Figure 3), the observed effect appears to be specifically related to enhanced migratory capacity.

In fibroblasts, by contrast, BzATP slightly enhanced migration at later time points, although the effect was less pronounced than in keratinocytes. This divergence in migratory responses between cell types may reflect differences in cytoskeletal organization, baseline motility, or downstream P2X7 signaling mechanisms. Notably, during extended incubation, BzATP may also degrade extracellularly into ADP and adenosine, which could engage additional purinergic receptors such as P2Y or adenosine (P1) receptors. These secondary effects are difficult to disentangle from direct P2X7 activation during long-term exposure. However, our study was designed to evaluate the overall impact of sustained P2X7 modulation on skin cell behavior rather than to isolate receptor-specific effects.

Unlike many previous studies that investigated P2X7 function in short-term assays or combined stimulation–inhibition protocols, our study focused on the sustained effects of pharmacological modulation using the agonist BzATP and antagonist A438079. This approach aimed to reflect potential in vivo conditions during prolonged exposure to purinergic signals—for example, during chronic inflammation or extended phases of tissue repair. In line with this aim, we did not investigate combined treatment with BzATP and A438079 as our goal was to evaluate the long-term, independent effects of either P2X7 activation or inhibition on skin cell behavior, rather than short-term receptor-specific blocking strategies. The concentrations of BzATP (300 µM) and A438079 (10 µM) were selected to ensure effective receptor modulation without inducing cytotoxicity. Both compounds were well tolerated by keratinocytes and fibroblasts, as confirmed by cell viability assays (Figure 4), indicating that these doses did not compromise cell survival under our experimental conditions. Similar concentrations have also been used in previous studies on epithelial wound healing in metazoan models [23]. Furthermore, the relatively short half-life of BzATP in aqueous media (30–60 min) supports the rationale for using higher concentrations in long-term incubations as even transient activation may induce sustained downstream signaling responses [17].

## 4. Materials and Methods

### 4.1. Tissue Collection and Cell Cultures

Skin fragments for immunofluorescence staining and cell isolation and cultures were obtained from six healthy female donors (aged 35–50) undergoing aesthetic breast reduction at the Department of Plastic Surgery, Medical Centre of Postgraduate Education, Orlowski Memorial Hospital in Warsaw. The study was approved by the Ethics Committee of the Medical Centre of Postgraduate Education, Orlowski Memorial Hospital in Warsaw (approval No. 63/PB/2016, dated 16 November 2016).

Keratinocytes and fibroblasts were isolated from skin explants according to the method described in detail by Guo et al. [24]. Briefly, the skin explants were cut into 1 mm^2^ fragments and placed into 6-well plates. After adhesion to the plate surface, the explants were covered with keratinocyte culture medium (KGM2, PromoCell, Heidelberg, Germany) and incubated at 37 °C. After approximately two days, primary keratinocytes growing out from the explants were collected by incubation with TrypLE™ Express (ThermoFisher Scientific Inc., Waltham, MA, USA) for 5 min at 37 °C. Fibroblasts migrating from the explants were collected after 6 days using the same TrypLE™ solution. Thus, obtained primary keratinocytes were propagated in KGM2 medium under standard cell culture conditions at 37 °C in a 5% CO_2_ humidified atmosphere. Dermal fibroblasts were propagated in Dulbecco’s Modified Eagle’s Medium (DMEM, ThermoFisher Scientific Inc.) supplemented with 10% fetal bovine serum (FBS) and 1% antibiotic–antimycotic solution (both from ThermoFisher Scientific Inc.). For both cell types, the medium was changed every second day, and cells were passaged as needed. All experiments were performed using cells at the third passage.

Matched keratinocytes and fibroblasts derived from the same donors (Patient IDs: 5, 37, 39, 56, 61, and 67) were used for further experiments. Cells isolated from all patients were included in the qRT-PCR analysis. For the proliferation, viability, and migration assays, cells from four donors (Patient IDs: 5, 37, 39, and 56) were used.

### 4.2. Proliferation and Viability Assays

To analyze cell proliferation, 100,000 keratinocytes or fibroblasts were seeded into 24-well plates (Nest Scientific Biotechnology, Wuxi, Jiangsu, China) in the respective culture medium and incubated for 24 h. Cells were then treated with 300 μM BzATP (Jena Bioscience, Jena, Germany; cat. No. NU-1620-25) or 100 μM A438079 (Tocris, Bristol, UK; cat. No. 2972/10) for 24 and 48 h. Untreated cells served as control. Following incubation, cells were detached using TrypLE™ Express (ThermoFisher Scientific Inc.) and analyzed for cell number and viability using the ADAM-MC™ image-based automated cell counter (NanoEntek, Seoul, Republic of Korea).

### 4.3. Wound Healing Assay Using Cell Culture Inserts

Cell migration was assessed using a wound healing assay with IBIDI culture inserts (IBIDI GmbH, Planegg, Germany), following the manufacturer’s instructions. A total of 14,000 keratinocytes or fibroblasts were seeded into each chamber of the insert and incubated overnight in KGM2 or DMEM supplemented with 5% FBS, respectively. After removal of the inserts, cells were cultured in the presence of 300 μM BzATP or 100 μM A438079. Untreated cells served as control. Cell monolayers were imaged every 6 h using the Spark^®^ multimode microplate reader (Tecan, Männedorf, Switzerland), and the wound healing area was quantified using ImageJ software, https://imagej.net/ (National Institutes of Health, Bethesda, MD, USA; accessed on 15 January 2025) [25].

### 4.4. Isolation of Total RNA and cDNA Synthesis

Total RNA was isolated using the Universal DNA/RNA/Protein Purification Kit (Eurx, Gdańsk, Poland) according to the manufacturer’s instructions. RNA concentrations were normalized, and cDNA was synthesized using the High-Capacity cDNA Reverse Transcription Kit (ThermoFisher Scientific Inc.) with oligo (dT) primers.

### 4.5. Quantitative Reverse Transcription PCR (qRT-PCR)

P2X7 gene expression was analyzed by qRT-PCR using an ABI 7500 FAST thermocycler (Applied Biosystems, Foster City, CA, USA), SensiFAST™ Probe Lo-ROX Kit (Bioline, Meridian Bioscience, London, UK), and specific TaqMan™ probes for P2X7 (Hs00951605_m1) and the reference gene TBP (Hs00427620_m1) (all from ThermoFisher Scientific Inc.). Relative mRNA levels were calculated using the ΔCt method with the Applied Biosystems™ Relative Quantification Software v4.3.

### 4.6. Western Blot Analysis

Keratinocyte and fibroblast lysates were prepared by homogenization in a lysis buffer containing 150 mM NaCl, 1% Triton X-100, 0.5% sodium deoxycholate, 0.1% SDS, and 50 mM Tris-HCl (pH 8.0). Protein concentrations were measured using the Pierce™ BCA Protein Assay Kit (ThermoFisher Scientific Inc.). Equal amounts of protein (20 µg) were separated by SDS–PAGE (10%) and transferred onto nitrocellulose membranes (ThermoFisher Scientific Inc.). After blocking with Pierce™ Protein-Free Blocking Buffer (Thermo Fisher Scientific Inc.), membranes were incubated overnight at 4 °C with primary antibodies: 1:1000 rabbit anti-P2X7 (cat. No. PA5-77665, ThermoFisher Scientific Inc.) or 1:3000 mouse anti-β-actin (clone AC-15, cat. No. A1978, Sigma-Aldrich, St. Louis, MO, USA).

Secondary HRP-conjugated antibodies (goat anti-rabbit IgG, cat. No. GTX213110-01; and rabbit anti-mouse IgG, cat. No. GTX213111-01; GeneTex, Irvine, CA, USA) were used and visualized with SuperSignal™ West Pico Chemiluminescent Substrate (ThermoFisher Scientific Inc.) on the ChemiDoc™ Imaging System (Bio-Rad, Hercules, CA, USA).

### 4.7. Immunofluorescence Staining

Skin specimens were snap-frozen in OCT Compound (Leica Biosystems Tissue Freezing Medium, cat. No. 14020108926, Wetzlar, Germany) and cryosectioned at 10 μm using a Leica CM1860 cryostat. In vitro cultured keratinocytes and fibroblasts were seeded on chamber slides (cat. No. 07-2101, Biologix, Jinan, China) and incubated for 24 h. Both cryosections and cultured cells were fixed in 4% paraformaldehyde for 20 min, followed by permeabilization in 0.5% Triton X-100 (Sigma-Aldrich). After washing with PBS (ThermoFisher Scientific Inc.), samples were blocked with 3% BSA in PBS for 30 min and incubated for 1 h with primary antibodies. For immunofluorescence staining of cultured cells, goat anti-P2X7 (SAB2501287, Sigma-Aldrich) and rabbit anti-SERCA2 (GTX55790, GeneTex) antibodies were used. For staining of skin tissue sections, rabbit anti-P2X7 (PA5-77665, ThermoFisher Scientific) and mouse anti-involucrin (clone SY5, MA5-11803, ThermoFisher Scientific) antibodies were applied. All primary antibodies were diluted 1:100. Secondary antibodies included Alexa Fluor™ 488 donkey anti-goat IgG (for goat anti-P2X7), Alexa Fluor™ 594 goat anti-rabbit IgG (for rabbit anti-SERCA2 and rabbit anti-P2X7), and Alexa Fluor™ 488 goat anti-mouse IgG (for mouse anti-involucrin) (all from ThermoFisher Scientific Inc.) used at 1:200 dilution. To determine immunostaining specificity, control samples were treated using only secondary antibodies. No non-specific signal was detected, indicating antibodies specificity.

### 4.8. Statistical Analysis

Statistical analyses were performed using GraphPad Prism 5 (GraphPad Software, La Jolla, CA, USA). Comparisons between matched keratinocyte and fibroblast samples derived from the same donors (Figure 2A) were assessed using the non-parametric Wilcoxon matched-pairs signed-rank test. For proliferation, viability, and migration experiments (Figure 3, Figure 4 and Figure 5), statistical significance between multiple experimental groups was analyzed using one-way analysis of variance (ANOVA) followed by Tukey’s post hoc test for multiple comparisons. Each time point was analyzed separately. A *p*-value < 0.05 was considered statistically significant.

## 5. Conclusions

Overall, our findings indicate that P2X7 modulates both growth and migration in human keratinocytes in a time-dependent and under different physiological conditions. This emphasizes the potential relevance of P2X7 in physiological processes such as wound healing, where sustained purinergic signaling may influence re-epithelialization dynamics. The differential responses of keratinocytes and fibroblasts to prolonged P2X7 stimulation or inhibition further support the idea that targeted modulation of this receptor could differentially affect distinct stages of skin repair.

## Data Availability

All relevant data are included within the manuscript. The raw data are available on request from the corresponding author.

## Abbreviations

The following abbreviations are used in this manuscript:

ATP	Adenosine 5′-triphosphate
ADP	Adenosine 5′-diphosphate
eATP	Extracellular ATP
DAMP	Damage-associated molecular pattern
